# Clinical presentations and long term prognosis of childhood onset polyarteritis nodosa in single centre of Korea

**DOI:** 10.1038/s41598-021-87718-6

**Published:** 2021-04-16

**Authors:** Jeong-Seon Lee, Joong-Gon Kim, Soyoung Lee

**Affiliations:** Department of Paediatrics, Seoul National University Children’s Hospital, 101, Daehak-ro, Jongno-gu, Seoul, 03080 South Korea

**Keywords:** Diseases, Medical research, Rheumatology

## Abstract

Childhood-onset polyarteritis nodosa (PAN) is a rare and systemic necrotising vasculitis in children affecting small- to medium-sized arteries. To date, there have been only a few reports because of its rarity. Thus, we aimed to investigate the clinical manifestations, laboratory findings, treatment, and long-term outcomes in patients with childhood-onset PAN and to evaluate the usefulness of the paediatric vasculitis activity score (PVAS). We retrospectively analysed the data of nine patients with childhood-onset PAN from March 2003 to February 2020. The median ages at symptom onset, diagnosis, and follow-up duration were 7.6 (3–17.5), 7.7 (3.5–17.6), and 7.0 (1.6–16.3) years, respectively. All patients had constitutional symptoms and skin manifestations, while five exhibited Raynaud’s phenomenon. Organ involvement was observed in one patient. The median PVAS at diagnosis was 7 (range: 2–32). Prednisolone was initially used for induction in all patients, and other drugs were added in cases refractory to prednisolone. All patients survived, but three patients with high PVAS at diagnosis experienced irreversible sequelae, including intracranial haemorrhage and digital amputation. In conclusion, early diagnosis and treatment may minimise sequelae in patients with childhood-onset PAN. This study suggests that high PVAS score at diagnosis may be associated with poor prognosis.

## Introduction

Polyarteritis nodosa (PAN) is defined as a necrotising inflammation of small- to medium-sized arteries without glomerulonephritis or vasculitis in the arterioles, capillaries, or venules that is not associated with antineutrophil cytoplasmic antibodies (ANCAs)^1^. Although epidemiological data related to childhood-onset PAN are relatively scarce, the disease is known to be more common in adults than in children^2,3^. Clinically, childhood-onset PAN presents as mild to severe inflammation, often leading to damage and early mortality^4^. The most recently published mortality rate for PAN in children is approximately 4% reported from the Great Ormond Street Hospital, and relapse rate was 35%^5^. Another large multicentre study from a tertiary referral centre, in which one-third of patients exhibited cutaneous forms of PAN, reported a mortality rate of 1.1%^6^. Early diagnosis and appropriate treatment for childhood-onset PAN are important for minimising irreversible complications and for improving prognosis^4^. Despite the presence of classification criteria, early diagnosis remains challenging because most patients with childhood-onset PAN visit the hospital due to non-specific constitutional symptoms. Myalgia and skin involvement are also significantly more frequent in patients with childhood-onset PAN than in those with adult-onset PAN^7^, which may lead to further delays in diagnosis. Moreover, PAN is difficult to treat due to a lack of guidelines, and only a few evidence-based studies have investigated childhood-onset PAN.

To assess the disease severity of PAN, the Five-Factor Score (FFS) was revised in 2009^8^. The paediatric vasculitis activity score (PVAS) has been also used to evaluate disease activity of PAN in many countries^9^. The PVAS is a clinical index of disease activity based on signs and symptoms, while the FFS is used for assessing prognosis and mortality. In previous studies, the five-year mortality rates were 46% and 12% in patients with an FFS 2 and 0, respectively^8,10^. However, neither instrument has been sufficiently evaluated in Korean paediatric patients with PAN.

The present study aimed to investigate the diagnosis, management, and outcomes of patients with childhood-onset PAN treated at a single tertiary centre in South Korea over a period of approximately 17 years. We further aimed to assess the efficacy of the PVAS and FFS in our patients.

## Results

### Clinical characteristics at diagnosis

Nine children (males: 6, females: 3) met the criteria of childhood onset PAN^11^ and were included in the present study. Most children visited the hospital with non-specific symptoms; other diagnoses were excluded, and diagnosis was confirmed through skin biopsy or angiography. The median age at initial signs or symptoms was 7.6 years (range: 3–17.5 years) and the median age at diagnosis was 7.7 years (range: 3.5–17.6 years). The median interval from onset of initial symptoms to diagnosis was 41 days (range: 30–249 days). The median follow-up duration was 7.0 years (range: 1.6–16.3 years). All patients were vaccinated against hepatitis B and had antibody levels expected to protect against the virus. None of the patients had a family history of rheumatic disease. The clinical manifestations and disease activity scores at diagnosis are summarised in Table 1. One patient was diagnosed with cutaneous PAN, while all others were diagnosed with systemic PAN. Skin signs were the most frequent initial manifestations of disease at diagnosis (purpura: 8, Raynaud’s phenomenon: 4, skin gangrene: 2, skin infarct: 2, subcutaneous nodules: 3, and livedo reticularis: 2), followed by musculoskeletal (arthralgia: 77.8%), systemic (fever: 77.7%), abdominal (abdominal pain: 22.2% and bowel ischaemia: 11.1%), neurological (headache, ptosis, stroke, and gait disturbance: 11.1%), and renal involvement (hypertension and serum creatinine elevation: 11.1%). None of the patients presented with mucous membrane, eye; ear, nose, and throat (ENT), chest; or cardiovascular symptoms. All patients were initially diagnosed with different diseases prior to the diagnosis of childhood-onset PAN. Five patients were diagnosed with joint problems such as reactive arthropathy, synovitis, psoriatic arthritis, or juvenile rheumatoid arthritis, three with unspecified vasculitis, and two with erythema nodosum. Thus, most patients (n = 8) had received oral or high-dose intravascular corticosteroids prior to the diagnosis of childhood-onset PAN. One patient received intravenous immunoglobulin G (IVIG) prior to PAN diagnosis for the treatment of truncal ataxia, left ptosis, and gait disturbance, resulting in improvement of symptoms (ptosis and truncal ataxia).

**Table 1:** Clinical characteristics at diagnosis.

	Patient 1	Patient 2	Patient 3	Patient 4	Patient 5	Patient 6	Patient 7	Patient 8	Patient 9
Sex	Male	Male	Male	Female	Male	Male	Male	Female	Female
Subtype	Systemic	Systemic	Systemic	Systemic	Systemic	Systemic	Systemic	Cutaneous	Systemic
Age at diagnosis (years)	17.6	5.8	5.6	3.5	7.5	9.4	7.7	15.3	11.1
Diagnosis delay (days)	30	30	62	192	31	89	41	249	32
**Initial symptoms at diagnosis**									
**General**									
Fever^a^	+	+	+	+	+	+	+	−	−
Weight loss^b^	+	−	−	−	−	−	−	−	−
Arthralgia	+	+	+	−	+	+	+	−	+
Myalgia	−	−	−	−	−	−	+	−	+
**Skin**									
Purpura	+	+	−	+	+	+	+	+	+
Subcutaneous nodules	+	−	+	−	−	−	−	−	+
Livedo reticularis	−	+	−	+	−	−	−	−	−
Skin infarct	−	−	−	−	−	−	+	−	+
Raynaud’s phenomenon	−	−	−	−	+	+	+	−	+
Gangrene	−	−	−	−	+	+	−	−	−
**Nervous system**									
Ptosis	−	−	−	+	−	−	−	−	−
Gait disturbance	−	−	−	+	−	−	−	−	−
Headache	−	−	−	+	−	−	−	−	−
Stroke	−	−	−	+	−	−	−	−	−
**Renal**									
Hypertension	−	−	−	+	−	−	−	−	−
Serum Cr↑^c^	−	−	−	+	−	−	−	−	−
**Abdominal**									
Abdominal pain	−	−	−	+	−	+	−	−	−
Bowel ischemia	−	−	−	+	−	−	−	−	−
Initial diagnosis	EN, Reactive arthropathy	Synovitis r/o JIA	Septic arthritis r/o vasculitis	Multiple sclerosis	Unspecified vasculitis	Unspecified vasculitis	Synovitis r/o JIA	EN	Psoriatic arthritis
FANA	−	−	−	−	−	−	−	1:320	−
ANCA	−	−	−	−	−	−	−	−	−
Other autoantibodies						Rheumatoid factor			
Steroid Hx^d^	+	−	+	+	+	+	+	+	+
Diagnostic method	Skin biopsy	Skin biopsy	Skin biopsy	Angiography	Skin biopsy	Skin biopsy	Skin biopsy	Skin biopsy	Skin biopsy
FSS	0	0	0	2	0	0	0	0	0
PVAS	6	6	4	32	9	13	7	2	7

EN: erythema nodosum; JIA: juvenile idiopathic arthritis; FANA: antinuclear antibody; ANCA: anti-neutrophil cytoplasmic antibody; FFS: Five-Factor Score; PVAS: Paediatric Vasculitis Activity Score.

^a^Documented axillary temperature, raised threshold to 38.0 °C.

^b^Loss of dry body weight without dieting ≥ 2 kg.

^c^Increase in serum creatinine > 30%, from 0.4 to 0.6 mg/dL.

^d^History, treated with prednisolone or methylprednisolone pulse therapy prior to the diagnosis of childhood-onset PAN.

**Figure 1:**
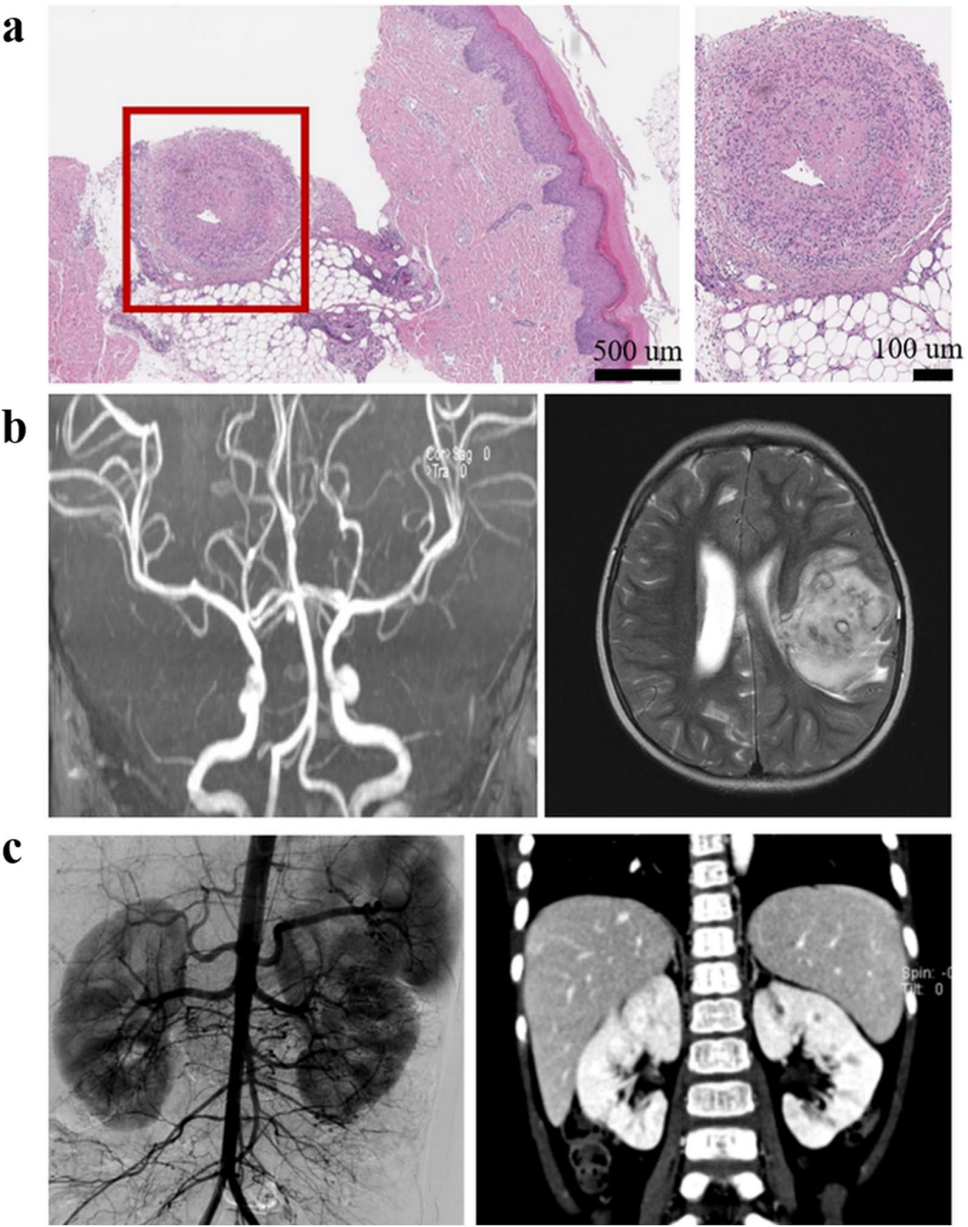
Pathologic and radiologic findings. (**a**) Histological findings for skin tissue: medium-sized vessels in the subcutis exhibiting lympho-histiocytic, some neutrophilic, and slight eosinophilic infiltration. (**b**) Brain arteriography showing small microaneurysms in the distal cerebral arteries and brain MRI showing brain infarction. (**c**) Ascending aortography showing multiple aneurysms and peripheral vascular luminal narrowing in the superior mesenteric artery, inferior mesenteric artery, and renal (bilateral), splenic, and right coronary arteries. Abdominal CT showing renal infarction and renal cortical thickening. MRI: magnetic resonance imaging; CT: computed tomography.

Five (55.6%) patients presented leucocytosis (median: 14,740/µL, range: 6960–31,290/µL), six (66.7%) patients presented thrombocytosis (median: 463,000/µL, range: 230,000–706,000/µL), and three (33.3%) patients had anaemia (median: 12.1 g/dL, range: 9.7–14.1 g/dL). All patients exhibited an increase in the erythrocyte sedimentation rate (ESR) (median: 48 mm/h, range: 9–125 mm/h), while seven patients (77.8%) exhibited increased high sensitivity C-reactive protein (hs-CRP) (median: 8.13 mg/dL, range: 0.01–28.63 mg/dL). No ANCAs were detected in any patient. Eight patients were diagnosed via skin biopsy. Perivascular lymphocytic infiltration was observed in seven patients (77.8%), neutrophilic infiltration was observed in three patients (33.3%), eosinophilic infiltration was observed in three patients, and fibrinoid necrosis (i.e., chronic PAN) was observed in one patient (11.1%) (Fig. 1a). Patient 4 was diagnosed via angiography because no inflammatory findings were observed in the skin, including in adipose tissue and vessels. In this patient, cerebral angiography and abdominal aortography revealed multiple microaneurysms in the distal cerebral, renal, and splenic arteries, which resulted in ischaemic damage in the brain (Fig. 1b) and kidneys (Fig. 1c). Since there were no suspected symptoms of streptococcal infection, Patient 8 was diagnosed as cutaneous PAN and was not evaluated for anti-streptolysin O (ASO) titre, produced by group A streptococcus bacteria.

The median PVAS at diagnosis was 7 (range: 2–32). Only one child (Patient 4) had an FFS of 2, while all others had FFS equal to 0.

### Treatment modality

All patients were initially treated with prednisolone. Table 2 outlines the initial treatment modalities and duration of induction and remission for all patients. In patients with mild organ involvement (i.e., peripheral neuropathy, cutaneous involvement without skin infarcts), low- to medium-dosed steroids represented the treatment of choice, occasionally in combination with azathioprine. In patients with severe, life-threatening multiple system involvement (i.e., severe skin or brain infarcts, brain haemorrhage), the treatment of choice consisted of high-dose intravenous (IV) administration of 6-methylprednisolone (mPd) (10–30 mg/kg, once daily for 3 days), followed by oral prednisolone^2^. Patients who did not respond to high-dose IV mPd and those with severe skin infarcts or brain involvement were treated with high-dose IV cyclophosphamide (600 mg/m^2^ body surface area) at 30-day intervals, and again with high-dose IV mPd, and/or IV infliximab (REMICADE, tumour necrosis factor alpha [TNF-α] inhibitor) at 5 mg/kg^12–14^, depending on individual toxicity profiles or physician preference at 14-day intervals. IVIG was administered based on the patient’s clinical status^15–17^. In six patients, other drugs were added for induction: cyclophosphamide in five patients, IVIG in two patients, and infliximab in one patient. Except for one child (Patient 8) who started the first treatment with the dermatologist, maintenance treatment was provided to the remaining patients who reached remission after induction. Although azathioprine (1–2 mg/kg), methotrexate (10 mg/m^2^ body surface area), or low-dose oral prednisolone represented the drug of choice in most patients, one child (Patient 6) was treated with cyclosporine instead, due to the side effects of oral prednisolone. Four children (Patients 5, 6, 7, and 9) initially presenting with Raynaud’s phenomena were treated with vasodilators such as nifedipine and IV alprostadil (EGLANDIN, prostaglandin E1).

**Table 2:** Initial treatment.

Patient	Induction	Maintenance	Induction duration (days)^a^	Remission duration (days)^b^
1	IV high dose mPd 1 g	AZT, PD	42	751
2	PO PD 15 mg	PD	217	240
3	PO PD 20 mg	PD	599	3866
4^c^^,d^	PO PD 25 mg IV high dose CPM 300 mg every month (6 doses)	PD	222	215
5^d^	IV high dose mPd 700 mg over 3 days (3 doses) PO CPM 75 mg IVIG	PD	297	163
6^d^^,e^	IV high dose mPd 900 mg IV high dose CPM 740 mg every one month (4 doses)	PD, Cyclosporin	225	161
7^d^	IV high dose mPd 750 mg IV high dose CPM 750 mg every one month (6 doses) IVIG Infliximab 170 mg every 2 weeks (15 doses)	AZT, MTX, PD	206	117
8	PO PD 30 mg	AZT	49	189
9^d^	IV high dose mPd IV high dose CPM 500 mg every one month	AZT, PD	81	210

Initial treatment of nine patients. IV: intravenous; mPd: 6-methylprednisolone; AZT: azathioprine; PD: prednisolone; PO: oral administration; CPM: cyclophosphamide; IVIG: intravenous immunoglobulin G; MTX: methotrexate.

^a^From the initial treatment to the start of steroid dose reduction.

^b^From the start of maintenance therapy to the last follow-up or to the first relapse.

^c^Patient 4 was treated with IVIG and several mPD pulse therapies before being diagnosed with PAN.

^d^Treated with nifedipine ointment and alprostadil for Raynaud’s phenomenon.

^e^Oral cyclosporin A was used for maintenance and was stopped because of hypertensive encephalopathy.

### Clinical manifestations during follow-up

The clinical manifestations observed during follow-up are presented in Table 3. During the follow-up (median, 7.0 years; range: 1.6–16.3 years), seven patients (77.8%) experienced relapse. The median time from remission to initial relapse was 163 days (range: 117–3866 days), and the median number of relapses was 1 (range: 0–3). Relapses were mostly minor and predominantly involved the skin in all six patients. In one child (Patient 3), if the steroid dose was slightly reduced after induction, skin symptoms and changes in laboratory results occurred; thus, it required 553 days to start maintenance treatment. Three patients experienced multiple relapses during the follow-up period. During the first induction treatment, the median duration of administration was 217 days (range: 42–557 days) (Table 2). However, during the first relapse, the median duration of induction treatment was 17 days (range: 0–29 days). Hence, the duration of hospitalisation was shorter during the first relapse. Raynaud’s phenomenon was identified in five of seven patients with relapse symptoms. During the initial treatment after diagnosis, two of four patients with Raynaud’s phenomenon necessitated amputation, although none required amputation due to Raynaud’s phenomenon that was caused by relapse. Patient 4 underwent several surgeries due to intracranial haemorrhage, which resulted in right-sided hemiparesis. No deaths were observed during the follow-up period (median: 7.0 years, range: 1.6–16.3 years).

**Table 3:** Major events during follow-up.

Patient	Follow-up, years	1st relapse, days	Number of relapses	Symptoms at relapse	Operation	Remaining symptoms	Adverse drug reactions	PVAS	
								First relapse	Last follow-up^f^
1	2.5	–	0	–	–	Hand tremor	Cushingoid face	–	0
2	1.6	–	0	–	–	–		–	0
3^a^	16.3	3866	2	Fever, oral ulcer, arthralgia, purpura, Raynaud’s phenomenon	–	Livedo reticularis		7	0
4	15.8	215	1	Abdominal pain Headache, vomiting	Brain surgery^b^	Subcutaneous nodule, Hemiplegia		5	1
5	12.0	163	2	Fever, skin nodule, Raynaud phenomenon	Amputation	Arthralgia	Hypertension	3	1
6^c^^,d^	9.9	161	1	Raynaud’s phenomenon	Amputation	–	Hypertension^c^, Pseudomembranous colitis^d^	1	0
7^e^	7.0	117	3	Raynaud’s phenomenon, Purpura	–	Subcutaneous nodule	Hypertension, Cushingoid face, Buffalo hump, haemorrhagic cystitis^e^	3	0
8	3.0	189	1	Purpura	–	–	–	2	1
9^c^	2.0	210	1	Raynaud’s phenomenon	–	Purpura	Cushingoid face	3	1

Major events during the follow-up for the nine patients.

^a^Patient 3 maintained high-dosed steroids due to worsening symptoms upon steroid reduction, and the period before entering maintenance treatment was 599 days.

^b^Craniotomy and intracerebral hematoma evacuation were done due to intracranial haemorrhage.

^c^Oral cyclosporin A was used for maintenance and was stopped because of hypertensive encephalopathy.

^d^Diarrhoea occurred 30 days after the start of steroid treatment, and was found to be due to pseudomembranous colitis.

^e^The first treatment as well as the treatments at the first recurrence and second recurrence included cyclophosphamide pulse therapy, and at the third recurrence, the patient developed haemorrhagic cystitis after the cyclophosphamide pulse therapy.

^f^PVAS was not scored as PVAS new/worse, but as PVAS persistent.

Adverse drug reactions included hypertension in three patients taking prednisolone. Of these, one experienced a seizure and exhibited proven hypertensive encephalopathy. Other adverse drug reactions due to prednisolone included Cushing syndrome, gastritis, fatty liver, osteoporosis, compression fracture, and infection. One patient experienced haemorrhagic cystitis due to cyclophosphamide treatment, which improved following discontinuation of the drug.

### Disease status at the most recent follow-up

At the most recent follow-up, two patients (Patients 5 and 6) were in clinical remission without medication, while seven patients were in clinical remission with prednisolone for maintenance therapy. In Patient 4, who had experienced multiple relapses and multi-organ involvement, the last relapse occurred in December 2013. She currently continues to take low-dose prednisolone, methotrexate, and azathioprine and is currently engaged in rehabilitation therapy.

## Discussion

To our knowledge, the present study is the first to report the clinical characteristics and long-term outcomes of childhood-onset PAN in South Korea and to analyse the utility of the FSS and PVAS. Given the partial agreement of our findings with those of previous reports, we believe that our study provides useful data related to the clinical manifestations of childhood-onset PAN.

Previous studies^6,18,19^ have reported that skin involvement and arthralgia/arthritis are more common in children than in adults with PAN. In contrast, weight loss and renal, gastrointestinal, and neurologic involvement may be more common in adults with PAN. In the largest multicentre study to date, which included 110 paediatric patients with PAN (63 with systemic PAN)^6^, 92% of patients exhibited cutaneous lesions, while 71.4%, 43%, 33.3%, 14%, and 11% exhibited myalgia, hypertension, central nervous system involvement, cardiac involvement, and pulmonary involvement, respectively. In another large paediatric study including 69 patients with systemic PAN^5^, the presentation of symptoms by organ system were as follows: skin involvement in 88%, myalgia in 83%, arthralgia/arthritis in 75%, weight loss in 64%, fever in 60%, renal involvement in 19%, severe gastrointestinal involvement in 10%, and central nervous system involvement in 10%. In accordance with previous findings, skin and joint symptoms were noted in most of our patients, although the rates of myalgia were lower. These findings suggested that cutaneous manifestations with non-specific systemic symptoms in children necessitate referral to a specialist in paediatric rheumatology or dermatology to ensure prompt clinical diagnosis.

Laboratory features including serum creatinine, urinalysis, liver function tests, and muscle enzyme concentrations were largely non-specific and included elevated ESR and hs-CRP in most cases. However, these findings are neither selective nor specific for the diagnosis of PAN. Currently, there are no laboratory findings that can be used to definitely diagnose either childhood- or adult-onset PAN. Evidence suggests that childhood-onset PAN affects girls more than boys, which is in contrast to adult-onset PAN^5,10,18^. Although we enrolled only a small number of patients, the prevalence of PAN appeared slightly higher in boys (n = 6) than in girls (n = 3). While previous studies have reported mortality rates ranging from 1 to 4%^5,6^, no deaths were observed during our study period.

We also evaluated PVAS and FFS in our patients. Three (Patient 4, 5, and 6) of the nine patients with the highest PVAS needed brain surgery or amputation. Although the total number of patients was small, those with a higher PVAS had poor prognosis. None of the patients presented involvement of the mucous membranes, eyes, ENT, chest, or cardiovascular system (used for PVAS assessment). The nervous system score of Patient 4, who had the most severe symptoms, was 25 points, which was a substantial difference from the maximum score of neurological symptoms. Skin manifestations, especially Raynaud’s phenomenon, with or without digital necrosis, were observed in four patients and five patients at diagnosis and at the first relapse, respectively. For the PVAS, Raynaud’s phenomenon is classified as other skin vasculitis and scored 1. Nonetheless, Raynaud’s phenomenon greatly influenced the treatment strategies adopted for our patients. Therefore, in the next PVAS revision, it would be better to consider the following: 1) adjustment of the maximum score relative to nervous symptoms and 2) classification of Raynaud’s phenomenon as a new category rather than being classified as another type of skin vasculitis and another factor to raise the allocation score. The highest FFS (2 points) was observed in Patient 4, who experienced hemiplegia due to cerebral bleeding and haematoma as well as several relapses. Fortunately, disease activity in Patient 4 has been well-controlled with low-dose prednisolone, azathioprine, and methotrexate. Although this finding indicates that the FFS may be useful for predicting mortality in both childhood- and adult-onset PAN, our study was limited by the small size of the study population. As few patients exhibited multi-organ involvement, it was difficult to assess the utility of the FFS in paediatric patients.

**Figure 2:**
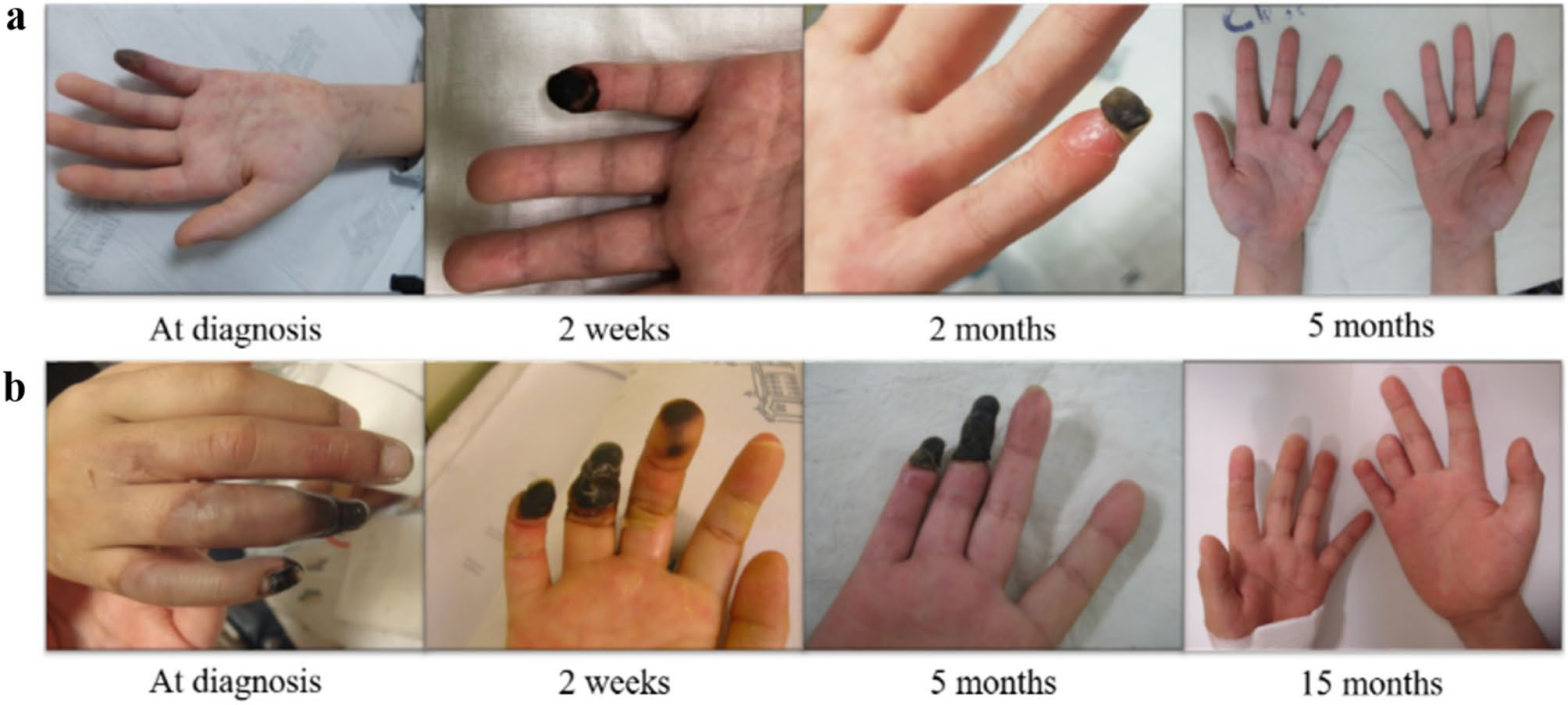
Treatment responses and final outcomes for patients with skin involvement. (**a**) This patient exhibited Raynaud’s phenomenon in the third, fourth, and fifth fingertips as well as skin necrosis in the fifth fingertip at diagnosis. Early diagnosis and aggressive treatment allowed for preservation of the fifth finger despite slight shortening. (**b**) This patient experienced finger pain at the initial visit and gangrene in the third, fourth, and fifth fingers, which worsened during transfer despite corticosteroid treatment. Despite aggressive treatment, amputation of the fourth and fifth fingertips was necessary.

The most appropriate treatments for PAN still remain to be determined. Given the rarity of the disease, no treatment guidelines have been fully established, especially for children. Herein, Raynaud’s phenomenon in the fingers was observed in four patients at diagnosis and in five patients at relapse. Finger amputation was necessary in two of these patients due to delayed diagnosis (Fig. 2a). In contrast, two patients in which Raynaud’s phenomenon had been detected earlier during the initial diagnosis and all patients whose symptoms had been recognised at the first relapse during the outpatient follow-up recovered without the need for amputation (Fig. 2b). These findings suggested that early diagnosis or detection and aggressive intervention helps to improve long-term outcomes. Initial treatment with corticosteroids is important in achieving remission in patients with adult-onset PAN, and steroid monotherapy is currently recommended for patients with mild PAN^20,21^. Moreover, cyclophosphamide may induce remission, and immunosuppressive agents such as azathioprine or methotrexate are recommended to maintain remission or as steroid-sparing agents^22^. For induction, all patients in the present study were treated with high-dose corticosteroids or corticosteroid pulse therapy. The degree of multi-organ involvement and the response to initial treatment were the principal determinants for the addition of cyclophosphamide, IVIG, or infliximab. Given the lack of controlled studies, IVIG treatment for vasculitis has been restricted to patients exhibiting resistance to corticosteroids and immunosuppressive therapy^23^. Both steroids and cyclophosphamide are associated with several side effects when used in children, including growth concerns, metabolic syndrome, adverse psychological effects, hypertension, infection, malignancy, and infertility^24–26^. In the present study, we also observed adverse drug reactions including a Cushingoid face (n = 3), hypertension (n = 3), infection (n = 2), osteoporosis (n = 1), and haemorrhagic cystitis (n = 1). One child (Patient 7) presented haematuria and was diagnosed with haemorrhagic cystitis. He received cyclophosphamide pulse therapy as an induction treatment at diagnosis, and at the first, second, and third relapses. Haemorrhagic cystitis occurred after the fourth cyclophosphamide pulse, which was improved only by hydration. These complications are particularly worrisome for children with many years of life ahead of them. Although we did not detect late-onset complications such as malignancy or infertility in our study, physicians should screen for signs of infertility and malignancy in patients exposed to cyclophosphamide.

Recent reports have highlighted the safety and efficacy of the TNF-α inhibitor in patients with PAN^27,28^. Infliximab appeared to be effective in treating skin necrosis in our study. Although not used in the current study, the interleukin-6 blocker tocilizumab has also shown promise for the treatment of vasculitis^29,30^. Strategies that aim to reduce the treatment burden as much as possible should be a major goal for physicians when attempting to achieve remission. Furthermore, paediatricians should closely monitor the growth and psychological status of patients with childhood-onset PAN as well as the long-term outcomes.

Although our study did not conduct any genetic testing, multiple cases of systemic and cutaneous PAN have been identified in some Georgian Jewish or German families, consistent with an autosomal recessive inheritance-induced mutation in the adenosine deaminase 2 (*ADA2*)-encoding gene *CECR1* (cat-eye syndrome chromosome region candidate 1)^4^. The discovery of this association between *ADA2* and vasculitis has led to the reconsideration of the diagnosis in most cases of childhood-onset PAN. Hence, it remains critical to evaluate family history and identify *ADA2* mutations when considering a diagnosis of PAN, as TNF-α inhibitors and *ADA2* replacement therapy may be warranted in some cases^28^.

This study has several strengths. First, all patients were managed by two expert physicians affiliated with the Seoul National University Children’s Hospital, who consulted to ensure homogenous referral and treatment patterns for all patients. Second, the patients were followed up for a relatively long period of time, allowing to better characterise disease status based on clinical and laboratory information. Third, although the study sample was limited, studies on PANs in childhood and adolescence are rare in South Korea. Despite these strengths, the study possesses some limitations of note, including its single-centre retrospective design, which prevented us from assessing correlations between various treatment strategies and final outcomes. We were unable to perform a comprehensive multivariate analysis including clinical manifestations, FFS, and PVAS due to the small population. Moreover, *DADA2* gene testing was not performed at the time of diagnosis. Lastly, we cannot exclude the potential for bias due to the inclusion of patients with more severe disease, who may be more likely to consult at tertiary referral centres. Therefore, our study population may not be fully representative of the overall situation at the regional or national level.

In conclusion, childhood-onset PAN is a rare disease that can be associated with severe complications despite appropriate treatment. Patients exhibiting central nervous system involvement, either at onset or during the disease course due to treatment, tend to experience the worst outcomes. Therefore, early diagnosis and aggressive treatment for childhood-onset PAN are important for improving prognosis without sequelae. Although our study provides some insight, additional multicentre studies are required to clarify the unique characteristics of childhood-onset PAN and to establish treatment guidelines, early detection strategies, and biomarkers.

## Methods

### Patients

All patients diagnosed with PAN who were treated in our department from March 2003 to February 2020, including those living outside our local catchment area, were eligible for this study. All patients were diagnosed prior to 18 years of age and satisfied both the American College of Rheumatology criteria^2^ and the European League Against Rheumatism/Paediatric Rheumatology International Trials Organisation/Paediatric Rheumatology European Society (EULAR/PRINTO/PRES) criteria^11^. In accordance with these criteria, patients were diagnosed with PAN when they presented with a systemic illness characterised by either a biopsy showing necrotising vasculitis (i.e. granulocyte or mixed leukocyte infiltrates in the arterial wall) in small- or medium-sized arteries or confirmed angiographic abnormalities (e.g., vascular aneurysms and occlusions). Additionally, patients were required to exhibit at least one of the following five clinical items: skin signs, myalgia/muscle tenderness, peripheral neuropathy, hypertension, or renal vascular disease. Cutaneous PAN was classified as follows: necrotising vasculitis of medium-sized arteries within the skin, without involvement of the internal organs, accompanied by fever, painful subcutaneous nodules, livedo reticularis, arthralgia, or myalgia^19,31^. The present study was approved by the Institutional Review Board of Seoul National University Hospital in South Korea (H-2004227–1119), who waived the requirement for informed consent because of the retrospective nature of the study.

### Data collection at diagnosis

Each patient’s medical records were reviewed in detail. From the time of diagnosis, the following data were retrieved: age; sex; the first clinical signs attributable to vasculitis; the time required to reach diagnosis (defined as the time from the first signs attributable to vasculitis until the date vasculitis was diagnosed via angiography or biopsy); signs of peripheral neuropathy; and manifestations of skin, central nervous system, genital, gastrointestinal, musculoskeletal, urologic, and renal involvement. We also collected data related to functional impairments such as fever (body temperature > 38.0 °C); weight loss, and malaise; laboratory findings including autoantibodies, viral serology, renal parameters (proteinuria, haematuria, and serum creatinine levels), and inflammation parameters (ESR and hs-CRP levels). We also reviewed initial treatments, responses to treatment, and histologic/radiologic findings.

### Definition of disease activity and damage

During diagnosis, disease activity was assessed using the PVAS, which was assigned retrospectively. The PVAS is a clinical index of 64 manifestations of active vasculitis allocated to one of nine organ-based systems^9^. The new/worse scale has a maximum score of 63, while the persistent scale has a maximum overall score of 33. Prognosis was assessed using the FFS^8^. FFS assessments are based on the following five items: renal insufficiency, proteinuria (> 1 g/day), central nervous system involvement, cardiac involvement, and severe gastrointestinal involvement (e.g., bleeding, infarction, and/or pancreatitis). Each item is assigned 1 point, and higher scores are considered indicative of increased mortality.

### Data collection during follow-up

During follow-up, we collected treatment-related data and recorded the timing from diagnosis to the first remission, the number of relapses, and the relapse symptoms. We also obtained photos reflecting changes in symptoms over time and re-assessed multi-system involvement (i.e., musculoskeletal, skin, cardiovascular, renal, gastrointestinal, peripheral vascular, neuropsychiatric). Comorbidities such as diabetes mellitus and other conditions such as haemorrhagic cystitis or proteinuria were also assessed. Remission was defined as the absence of any clinical signs/symptoms of active vasculitis, as supported by laboratory evidence of normal CRP and ESR values and a PVAS of 0 of 63 (assigned retrospectively for every clinic visit), for 2 evaluations at least 1 month apart and with adherence to the prednisolone regimen. Relapse was defined as the reoccurrence of PAN manifestations in a patient whose disease had previously been in sustained remission for 3 months and required the addition of or a change in immunosuppressive agents, reinstitution of corticosteroids, and/or an increase in dose (dosage increased by > 50% to > 0.5 mg/kg/day).

### Statistical analyses

Categorical variables are expressed as numbers (%), while continuous variables are expressed as medians (range: minimum–maximum) or the mean ± standard deviation. All statistical analyses were performed using IBM SPSS Statistics ver. 25.0 (IBM Co., Armonk, NY, USA).

### Ethics approval and consent to participate

This study was conducted in accordance with the Declaration of Helsinki. The Institutional Review Board of Seoul National University Hospital in South Korea approved the research protocol (H-2004227-1119).

### Consent for publication

The Institutional Review Board of Seoul National University Hospital in South Korea, approved this study and waived the requirement for informed consent because of the retrospective nature of the study.

## Data Availability

The datasets used and/or analysed during the current study are available from the corresponding author on reasonable request.
